# “The Last of Them”: Entomopathogenic Effect of *Akanthomyces muscarius* on the Scale Insect Pest *Toumeyella parvicornis* Under Laboratory Conditions, a Potential Biological Control Candidate

**DOI:** 10.1111/ppl.70533

**Published:** 2025-09-20

**Authors:** Nicolò Di Sora, Silvia Turco, Federico Brugneti, Flavia Isoli, Angelo Mazzaglia, Mario Contarini, Stefano Speranza, Luca Rossini

**Affiliations:** ^1^ Dipartimento di Scienze Agrarie e Forestali Università degli Studi della Tuscia Viterbo Italy; ^2^ Centro de Estudios Parasitológicos y de Vectores (CEPAVE, CONICET‐UNLP) La Plata Argentina; ^3^ Service d'Automatique et d'Analyse des Systèmes Université Libre de Bruxelles Brussels Belgium

**Keywords:** arthropod pathogens, beneficial agents, biocontrol, tortoise scale insect, urban pest

## Abstract

Entomopathogenic fungi are valuable alternatives to traditional agrochemicals, providing more sustainable crop protection. *Akanthomyces* spp. is acknowledged in biocontrol practices, especially in controlling aphids, thrips, whiteflies, and mites; however, its efficacy on soft scale insects is still poorly known. This study investigates the potential use of *Akanthomyces muscarius* on the soft scale insect *Toumeyella parvicornis*, a new invasive pest for European stone pines ( *Pinus pinea* ). The strain of *A. muscarius* tested in the experimentation has been isolated from *Parthenolecanium corni*, a soft scale insect on which the fungus seems to be highly infective. After molecular identification and characterization, bioassays were conducted to compare the performances of the different conidial concentrations of *A. muscarius* isolate and commercial formulations on *T. parvicornis* overwintering females and nymphs. Results showed a promising colonization and higher pathogenicity of *A. muscarius* on *T. parvicornis* in all the trials, compared with the other commercial formulations, in some cases showing a quick infection and death of the host. Our results pave the way to further field uses where *A. muscarius* is applied as a biological control method to reduce infestations of *T. parvicornis* .

## 1 Introduction

The tortoise scale insect *Toumeyella parvicornis* (Cockerell) (Hemiptera: Coccidae) is rapidly becoming the main pest of stone pine plants ( *Pinus pinea* L.) in Europe (Garonna et al. 2018; Di Sora et al. 2022). This pest is native to North America (Hamon and Williams 1984), and it is rapidly spreading in Europe, where, so far, it has been first reported in Italy (Garonna et al., Garonna et al. 2015), France (EPPO 2021), and Albania (Di Sora, Contarini, et al. 2024).

This insect pest is causing diebacks of stone pines mainly in urban areas, leading to a significant modification of the landscape (Di Sora et al. 2022; Di Sora, Mannu, et al. 2023; Di Sora, Rossini, et al. 2023) and high risks for park users and public security in general. Additionally, control actions in urban areas are strongly limited for safety reasons (Di Sora et al. 2022; Di Sora, Rossini, et al. 2023); hence, only endotherapic treatments with abamectin as the active ingredient are currently allowed. Despite the advantage of a limited spread of the agrochemicals in the environment, endotherapy is expensive, has a limited time coverage (Di Sora, Mannu, et al. 2023), and is unsuitable for large‐scale treatments in natural environments. Moreover, different national and international authorities will establish increasingly restrictive regulations on pesticide use, supporting more sustainable strategies applied in agricultural, urban, and forest environments (Schneider et al. 2023). Valuable alternative means of control involve the use of predators and/or parasitoids (Howarth 2003; Van Driesche et al. 2010; Di Sora, Rossini, et al. 2024), nematodes, or entomopathogenic bacteria, viruses, and fungi (Lacey et al. 2015; Hernández‐Rosas et al. 2020).

Entomopathogenic fungi are known for their peculiar relationship with arthropods (Shah and Pell 2003), for targeting a restricted group of hosts (or even a single species) (Shah and Pell 2003; Erdos et al. 2021), and for their endophytic adaptations (Nicoletti and Becchimanzi 2020). The selectivity of these natural enemies is a great advantage from a pest management point of view since, unlike conventional agrochemicals, it might preserve non‐target species (Bilgo et al. 2018; Shah and Pell 2003). According to the current literature, there are around 750 species of fungi with entomopathogenic effects on terrestrial arthropods, namely insects and mites (Sinha et al. 2016). The infection process is generally the following: (1) asexual spores (i.e., conidia) reach insect cuticle and germinate, endorsed by proteases, chitinases, and lipases enzymes and by suitable environmental conditions, (2) the mycelium penetrates the haemocoel, favored by penetration structures such as penetration pegs and/or appressoria, and supported by hydrolytic enzymes (Ortiz‐Urquiza and Keyhani 2013), (3) colonizes the overall body of the insect, feeds on hemolymph and secretes toxins (i.e., beauvericin), and leads individuals to death in a range of time approximately near to 3–4 days, in the most virulent cases. The latter parameter depends on the eventual insect immune responses, such as the release of antimicrobial substances, melanization, or even grooming behavior (Ortiz‐Urquiza and Keyhani 2013). After the host dies, the reproductive structures of the fungus come out from the body, spreading the spores in the surrounding environment (Samson et al. 1988; Shah and Pell 2003; Sinha et al. 2016).

Among the entomopathogenic fungi, *Akanthomyces* deserves particular attention because different isolates with lethal effects have been identified from a plethora of organisms, such as insects and mites (Lopes et al. 2023; Zare and Gams 2001), plants (Nicoletti and Becchimanzi 2020), and fruits (Roxana et al. 2019). *Akanthomyces muscarius* (Evan and Hywel‐Jones 1997) belongs to the family Cordycipitaceae, order Hypocreales, which is also reported with the synonyms *Verticillium lecanii*, *Lecanicillium lecanii*, or *L. muscarium* (Zare and Gams 2001).

The entomopathogenic effects of *A. muscarius* have already been investigated on different insect pests such as *Thaumetopoea pityocampa* (Denis & Schiffermüller) (Lepidoptera: Notodontidae) (Saidi et al. 2023), *Bemisia tabaci* (Gennadius) (Hemiptera: Aleyrodidae) (Broumandnia et al. 2021), and *Myzus persicae* (Sulzer) (Hemiptera: Aphididae) (Erdos et al. 2021). Recently, *A. muscarius* was also found in hazelnut galls infested by *Phytoptus avellanae* Nalepa and *Cecidophyopsis vermiformis* (Nalepa) (Turco, Drais, et al. 2024). The high suitability of *A. muscarius* as a biological control agent of insect pests has, in recent years, led to the marketability of different formulations to control whiteflies, thrips, aphids, and mites (De Faria and Wraight 2007; Bardin et al. 2008).

Although Evan and Hywel‐Jones (1997) included *A. muscarius* among the possible pathogens of soft scale insect species, the information is nowadays related only to a few species belonging to the *Eulecanium* and *Parthenolecanium* genera, neglecting the majority of Coccidae. This topic deserves an in‐depth investigation through specific and extensive studies, given the high number of soft scale insect pest species belonging to the family of Coccidae (Pellizzari and Germain 2010).

*Toumeyella parvicornis* is a good case study to assess the entomopathogenicity of *A. muscarius* , as this species still needs more decisive containment strategies. The detection of an isolate of *A. muscarius* from specimens of *Parthenolecanium corni* (Bouché, 1844) (Hemiptera: Coccidae) retrieved in a hazelnut field in the Viterbo area (Lazio, Central Italy) during a standard monitoring activity (Mazzaglia et al. 2023) was the backbone for the development of this study. After preliminary morphological and molecular characterisation, indeed, the research aimed to: (1) test under laboratory conditions the capability of the isolate to infect, colonise, and kill different life stages of *T. parvicornis* , (2) compare its control efficacy with commercial formulations already available on the market, and (3) to hypothesise its use in a biocontrol strategy against *T. parvicornis* .

## 2 Materials and Methods

### 2.1 First Samples of the Infected Scale Insect and Background Information From Natural Environments

**FIGURE 1:**
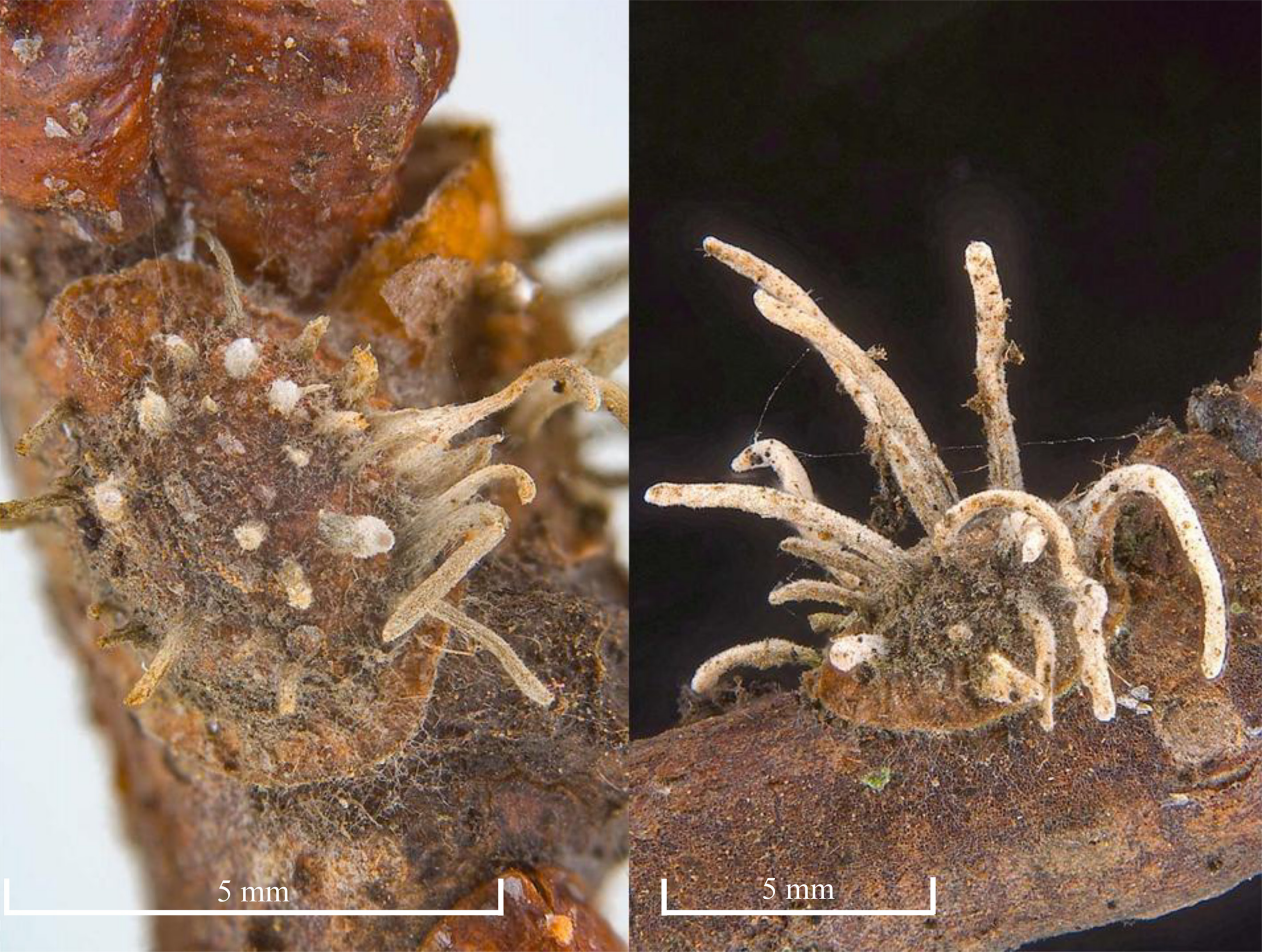
Hazelnut twigs infested by scale insects showing fungal fruiting bodies emerging from adult females.

In June 2023, a standard monitoring campaign was carried out in a hazelnut ( *Corylus avellana* L.) orchard located in Bassano in Teverina (Lazio, Central Italy—42°27′14.9″ N 12°17′58.0″ E, elevation: 306 m above sea level). During the activities, several scale insects with different life stages were observed on the smaller twigs of the plants. Additionally, from the bodies of many of these individuals, fungal structures were coming out, as shown in Figure 1. Randomly infested twigs with the same features as in Figure 1 were cut with pruning scissors and sealed in plastic bags for further inspection in the laboratory. The morphological analysis was carried out following the guidelines of Kondo and Watson (2022) for the adults and of Miller and Williams (1990) for the preimaginal stages, while the molecular characterization was based on the protocol of Di Sora, Rossini, et al. (2023) (DNA extraction and cytochrome oxidase subunit I (COI) PCR amplification).

### 2.2 Isolation and Morphological Identification of the Entomopathogenic Fungus Retrieved From Scale Insects

The morphology of the fungal fruiting bodies emerging from scale insects was noted and documented. Then, some random structures were aseptically collected, superficially sterilized by 70% ethanol washing (30 s) and subsequent rinsing in sterile water, plated on Potato Dextrose Agar (PDA 39 g L^−1^, Dextrose 20 g L^−1^, Potato extract 4 g L^−1^, Agar 15 g L^−1^, pH 5.6 approx Dinkelberg), and incubated at 25°C, 100% RH, in the dark. Single pure cultures were obtained by re‐isolating plugs from mixed colonies into fresh PDA plates. The morphological features of the colonies (shape and color) were observed for 10 days. The shape and dimensions of phialides and conidiogenous cells were recorded and measured. The mean length and width of 30 randomly selected conidia for each colony were measured with a 40× magnification microscope (Leitz).

### 2.3 Molecular Identification and Phylogenetic Analysis of Entomopathogenic Fungus Retrieved From Scale Insects

Genomic DNA was extracted from 100 mg of fresh pure mycelium using the NucleoSpin Plant II Midi DNA extraction kit (Macherey‐Nagel). Molecular identification of 2 randomly selected fungal isolates was carried out through Internal Transcribed Spacer (ITS) amplification using the ITS1‐ITS4 primers (White et al. 1990) and the following PCR protocol: initial denaturation at 95°C for 120 s followed by 35 cycles of 30 s at 95°C, 30 s at 57°C, and 30 s at 72°C, and a final elongation at 72°C for 5 min. The DNA amplicon was visualised on a 1.2% agarose gel and sent to Eurofins Genomics (Konstanz, Germany) for Sanger sequencing.

A more detailed phylogenetic analysis and taxonomic classification was carried out on one selected fungal strain, in addition to the ITS, by amplifying and sequencing also portions of the Large Ribosomal RNA Subunit (LSU), the Small Ribosomal RNA Subunit (SSU), the Elongation Factor (EF‐1α), the DNA‐directed RNA polymerase II largest (RPB1), and second largest (RPB2) subunits. The primer sequences and the PCR protocols used for the molecular identification are listed in Table S1.

The corresponding sequences of six genes (ITS, LSU, SSU, EF‐1α, RPB1, and RPB2) for the genetic similarity comparison were retrieved from the genomes of entomopathogenic fungi belonging to either *Akanthomyces, Lecanicillium*, or *Simplicillium* genera and from a few representatives of *Beauveria*, *Cordyceps*, and *Salmoniella* available in the NCBI database (Table S2).

The single sequences were visually adjusted using UGENE v48.1 (Okonechnikov et al. 2012), manually concatenated, and aligned with MUSCLE v3.8.31 (Edgar 2004). A maximum likelihood (ML) phylogenetic tree was obtained using RAxML‐HPC v8.2.12 (Stamatakis 2014), set with the GTRCATI algorithm as a substitution model and 1000 bootstraps. The tree was visualised using FigTree v1.4.4 (http://tree.bio.ed.ac.uk/software/figtree/) and edited with Inkscape v0.92 (www.inkscape.org).

### 2.4 Collection of *Toumeyella parvicornis* Specimens and Pathogenicity Tests

Presuming a similar effect of the fungus on *Toumeyella parvicornis* as observed on the specimens collected in the hazelnut orchard, and to verify this hypothesis, a set of laboratory experiments was set up, as detailed in the following sections.

#### 2.4.1 *T. parvicornis* Specimens and General Testing Conditions

Fresh stone pine twigs infested by *T. parvicornis* were collected in three suburban areas of Rome [Italy, 41°44′29.8″ N, 12°19′44.1″ E, elevation: 0 m above sea level (a.s.l.), 42°00′56.1″ N, 12°28′26.6″ E, elevation: 73 m a.s.l. and 42°07′23.2″ N 12°21′51.2″ E, elevation: 257 m above sea level (a.s.l.)], sealed in plastic bags and immediately brought to the laboratory. The number of adult females in the samples varied according to the seasons; subsequently, the number of adult females considered during the trials also varied. It is worth clarifying that, where possible, the number of specimens per twig has been kept comparable.

Tests were carried out under controlled conditions of 25°C ± 2°C, 70%–75% of relative humidity, and complete darkness. After the inoculation, pictures of individual symptoms were taken using a Leica Ergo Transmitted Light Base Camera (Model TL5000—DMC 5400—M205C).

#### 2.4.2 Preliminary Observations in Petri Dish Condition Tests on *T. parvicornis* Specimens

The conidial suspension was freshly prepared prior to each experiment by pouring conidia scraped from the mycelium into sterile distilled water (SDW). The suspension was subsequently filtered to remove larger fragments, quantified with a hemocytometer, and diluted to the final concentration according to the purpose of the investigation. Finally, it was sprayed on stone pine twigs.

Infested stone pine twigs were monitored once per day for 8 days after the inoculation to assess the presence of fungal structures on the specimens. After their detection, fungal structures were observed, re‐isolated, and identified as described in Section 2.2. At the end of the experiment, independently of the presence of the fungus, the vitality of each specimen was verified by piercing the body with a needle (from N2 to adults) or by observing the discoloration of the body (only for crawlers). The number of insects per treatment showing fungal structures was considered a quantitative variable.

Two life stages were selected as targets for the experimentation: overwintering females and the first nymphal stage (crawlers). Overwintering females are responsible for the infestation of the following year; moreover, they are present in natural environments when the conditions of relative humidity and temperature are favorable for fungal growth. Crawlers are responsible for spreading the outbreak in new environments and are less protected by wax structures; they can potentially be easily infected while spreading the fungus in the environment.

In a first test, 0.5 mL of *A. muscarius* NOC1 conidial solution at different concentrations, namely 10^4^, 10^6^, and 10^8^ conidia/mL, was sprayed on overwintering adult females of *T. parvicornis* attached to stone pine twigs (6.5 cm long and 0.5 cm diameter). Solutions have been double‐sprayed (front and reverse) from a distance of 15 cm from five twigs per treatment and sealed with parafilm in sterile Petri dishes (9 cm in diameter), while water was used as a control, maintaining standardized conditions. Once the most effective NOC1 conidial concentration (10^8^ conidia/mL) was identified, 0.1 mL was sprayed (from 15 cm of distance) on crawlers in Petri dishes. In parallel, Mycotal (*Lecanicillium muscarium* Ve6 = 1 mL of powder per liter of water, for a final conidial concentration of 10^7^, according to the Manufacturer's instructions) and distilled water were sprayed as a comparative test and as a control, respectively. Solutions were sprayed (from 15 cm of distance) on crawlers that were distributed in Petri dishes as follows: ten alive crawlers per Petri dish (handled using a moist brush), for a total of five dishes per treatment (200 crawlers in 20 Petri dishes), sealed with parafilm. Two pairs of pine needles per dish ensured a feeding substrate during the test.

#### 2.4.3 Pathogenicity Bioassay: Simulated Natural Condition Test on Overwintering *T. parvicornis* Females

In November 2024, 108 stone pine twigs (15–20 cm in length) infested by overwintering females were randomly collected from the field (Section 2.4.1) using pruning scissors, sealed in plastic bags, and brought to the laboratory for downstream experiments.

Twigs were individually placed into glass vials (5 cm long and 1 cm in diameter), previously half‐filled with sterile water to enhance tissue conservation over time. Vials have been subsequently inserted into plastic boxes (24 cm large, 33 cm long, and 8 cm high), provided with a polystyrene base for their support. Nine vials were placed in each box, for a total of 12 plastic boxes.

Each treatment involved groups of 4 boxes, for 3 treatments: *A. muscarius* NOC1 conidial solution at 10^7^ conidia/ml, Mycotal, and untreated control with distilled water. According to this protocol, every treatment was tested on 36 infested twigs. Every twig was covered, on average, by 7 overwintering adult females of *T. parvicornis* for overall 251, 251, and 254 adult females tested, respectively, for *A. muscarius* NOC1, untreated control, and Mycotal treatments. At the beginning of the experimentation, each twig was sprayed until dipping (saturation of the solution) with the associated conidial solution (~11 mL per twig) and stored inside the dedicated box (Table 1). Samples were checked daily for the first 8 days after the treatment and then, regularly, twice a week.

**TABLE 1:** Summary of the treatments and the methodologies applied during the pathogenicity bioassays.

Methodologies and parameters	Treatments		
	*A. muscarius* NOC1	Mycotal	Untreated control (water)
No. of stone pine twigs	36	36	36
Average no. of adult females per twig	~7	~7	~7
No. of stone pine twigs per plastic box	9	9	9
Conidial concentration	10^7^ conidia/mL	10^7^ conidia/mL	0
Applied dose	~11 mL per twig	~11 mL per twig	~11 mL per twig
Application method	Spraying until saturation	Spraying until saturation	Spraying until saturation

*Note:* The treatments: *A. muscarius* NOC1, Mycotal, and Untreated control (water). Among the methodologies and parameters are the number of stone pine twigs, the average number of adult females per twig, the number of stone pine twigs per plastic box, conidial concentration, applied dose, and application method.

Insects covered intact twigs under conditions simulating high humidity percentage and stable temperature. Every 2 days, fresh sterile water was vaporized on the samples to ensure proper humidity level inside the boxes and to maintain, as much as possible, the longevity of the samples. Moreover, the water inside the vials was changed, and a short portion of the twigs' basal section was removed to avoid any microbial proliferation. Adult females colonized by either NOC1 or Mycotal were recorded during the inspections. During the last day of the experimentation (Day 22), the total number of dead or alive adult females was noted down. Part of the specimens showing symptoms was collected and plated on a PDA substrate to verify the identity of the microorganism and confirm the colonization by the natural and the commercial formulation of *A. muscarius* (Section 2.2).

### 2.5 Data Analysis

Data were analyzed through the R software (R Development Core Team 2022), as detailed below. The complete dataset and the scripts to fully reproduce the results of the present study are publicly available at (https://github.com/nicodisora/aka).

#### 2.5.1 Analysis of the Dataset of Overwintering Females and the Crawlers

The dataset of the overwintering adult females was analyzed in three steps; the crawler's dataset was analyzed in two steps. Statistical differences between the overwintering adult females showing evidence of *A. muscarius* infection (or dead) and crawlers showing lethal symptoms among the different treatments were analyzed through a Generalized Linear Model (GLM) and the Dunnett adjusted as a *post hoc* test (α = 0.05). The same analysis was carried out on the complete dataset, referring to the overall days, and then singularly comparing the data collected daily. The analysis was carried out through the *glm()* function within the R package MASS (Ripley et al. 2013), while the *post hoc* test and pairwise comparison were conducted through the *emmeans()* function within the R package *emmeans* (Lenth and Lenth 2018), the *pairs()* function within the R package *multcompView* (Graves et al. 2015), and the *cld()* (Hothorn et al. 2016). The treatment was considered as a factor, while the number of *T. parvicornis* or crawlers infected or dead by different pathogenic fungi mycelium was a numerical value.

## 3 Results

### 3.1 Identification of *P. corni*

The morphological and molecular characterization (GenBank accession number PP778664) ascertained that the scale insect species retrieved on hazelnut twigs and presenting the mycological structures was *P. corni* (Supporting Information S1).

### 3.2 Isolation and Identification of *A. muscarius*

**FIGURE 2:**
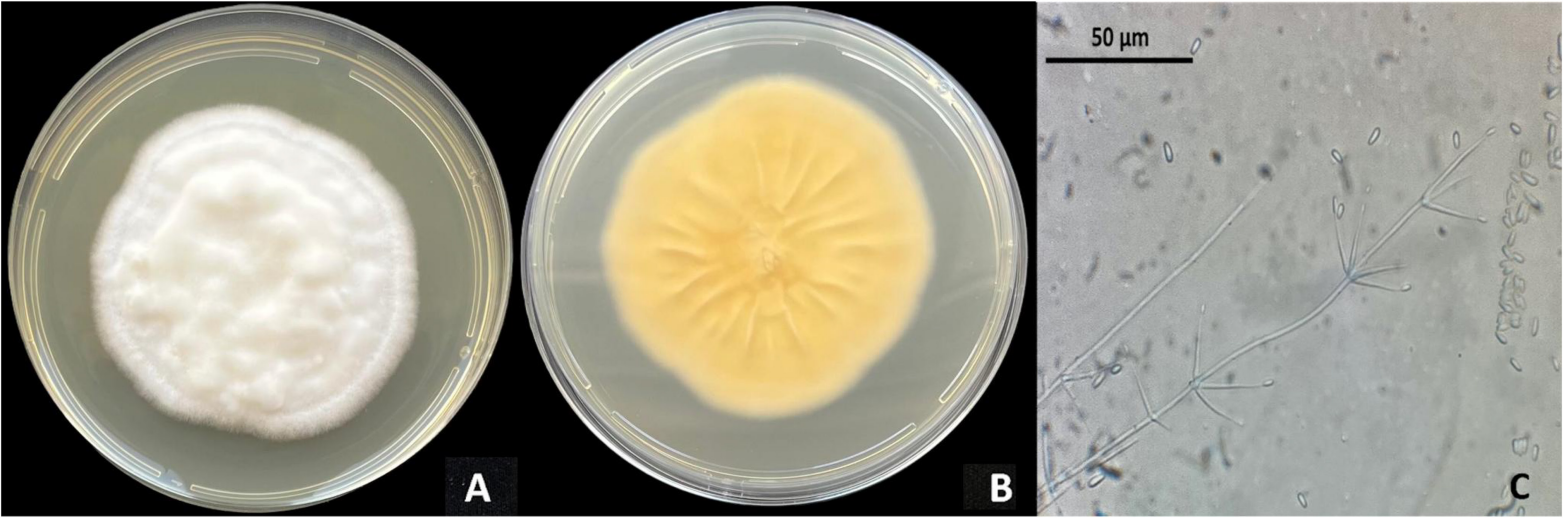
Front (A) and back (B) view of a 10‐day‐old PDA plate, phialides and conidia (C) of the isolate *Akanthomyces muscarius* NOC1.

After 10 days of incubation at 25°C, the mycelium on the PDA plates was compact, fluffy, white colored on the front and pale yellow on the reverse (Figure 2A,B). The phialides were erect, mostly punch‐shaped, and ranged from 22 to 42 μm. The conidia were hyaline and unicellular, with a subcylindrical, cylindrical, and ellipsoidal shape, and with a size that ranged from 2.2 to 7.2 μm in length and 1.5 to 2.8 μm in width (Figure 2C). According to Zare and Gams (2001), these morphological traits are typical of *A. muscarius* .

The morphological characterization was further confirmed by molecular analysis on one randomly selected isolate, “NOC1.” After DNA extraction, ITS amplification and sequencing, the isolate showed a 99.82% overlap with *A. lecanii* and *A. muscarius* . The sequences of the five additional genes (LSU, SSU, EF‐1α, RPB1, and RPB2), together with the ITS, were deposited in the NCBI database under the accession numbers OR730828‐30 (ITS, SSU, and LSU, respectively) and OR735523‐25 (EF‐1α, RPB1, and RPB2, respectively).

### 3.3 Phylogenetic Analysis

**FIGURE 3:**
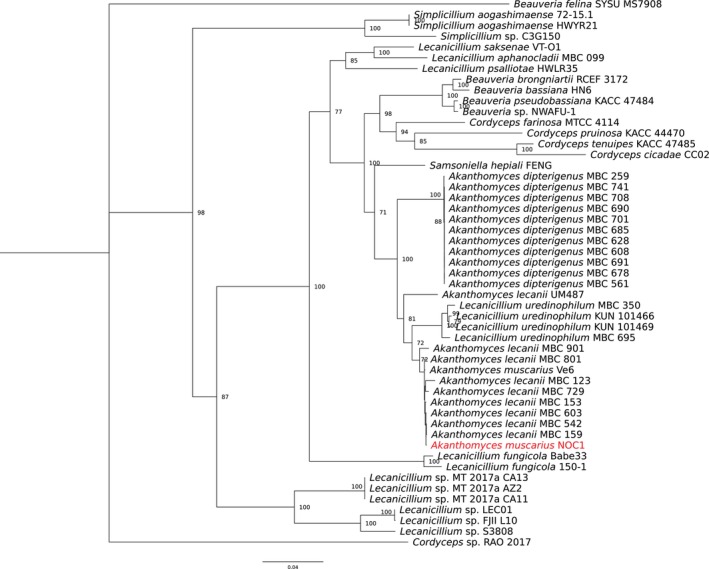
Maximum Likelihood (ML) phylogenetic tree on the concatenated sequences of six housekeeping genes among the Cordycipitaceae isolates under comparison. The NOC1 isolate (in red) is perfectly clustered within the *Akanthomyces* clade, together with the *A. dipterigenus* and closer to *A. lecanii/muscarius* isolates and *L. uredinophilum* isolates. The tree was rooted on *Cordyceps* sp. RAO 2017 and only bootstraps higher than 70 are shown.

The phylogenetic relationship of NOC1 with other Cordycipitaceae is shown in Figure 3. The isolate NOC1 perfectly fits within the *A. muscarius* clade, and other *A. lecanii* are close to the *A. uredinophilum* strain isolated from aphids and insects (BioProject PRJNA879330; Wei et al. 2018). The higher branches of the phylogenetic tree include *Salmonella hepiali* (also known as *Paecilomyces hepiali*), some isolates belonging to *Beauveria* (i.e., *B. brognartii*, *B. bassiana*, *B. pseudomonassiana*, and *Beauveria* sp.), *Cordyceps* (i.e., *C. farinosa* , *C. pruinosa* , *C. tenuipes* , and *C. cicadae*), and *Lecanicillium* (*L. saksenae*, *L. aphanocladii*, and *L. psalliotae*) genera. Other *Lecanicillium* sp. isolates, as well as *Simplicillium* isolates and *B. felina*, were slightly more distant, while the tree branch was rooted on *Cordyceps* sp.

### 3.4 Preliminary Observations From Petri Dish Condition Tests on *T. parvicornis* Specimens

One day after the beginning of the test, the first evidence of mycelium growing on the overwintering adult female specimens was observed. The days after, the mycelium spread from the marginal part of the body to the surrounding areas of the twig as well. On day eight, the mycelium was present on 92.60% of the adult females placed in the Petri dishes. However, only the concentrations 10^6^ and 10^8^ were statistically different from the untreated control (Table 2). Regarding dead adult females (Supporting Information S2), the highest value was observed at a concentration of 10^8^, which was statistically different from either 10^4^ or the untreated control (Table 2). Moreover, it is worth noting that only the 10^8^ suspension caused the death of 60% of the initial set of individuals involved in the test (8 days after the treatment application). Conversely, the deaths were around 26% with the 10^6^ suspension and 4% with the 10^4^ suspension.

**TABLE 2:** Statistical analysis was tested on *T. parvicornis* overwintering adult females and on crawler specimens by comparing the different treatments.

Preliminary observations	Treatment comparison	Mycelium on the body		Dead specimens	
		*t. ratio*	*p*	*t. ratio*	*p*
Overwintering adult females	10^4^ vs. control	−0.648	0.92	0.309	0.99
	10^6^ vs. control	2.916	0.01 < *p* < 0.05	2.160	0.18
	10^8^ vs. control	2.916	0.01 < *p* < 0.05	4.938	*p* < 0.001
	10^4^ vs. 10^6^	−0.648	0.92	−1.852	0.30
	10^4^ vs. 10^8^	2.268	0.15	−4.629	0.001 < *p* < 0.01
	10^6^ vs. 10^8^	0	1	−2.777	0.06
Crawlers	Mycotal vs. control			4.060	*p* < 0.001
	*A. muscarius* NOC1 vs. control			8.120	*p* < 0.001
	Mycotal vs. *A. muscarius* NOC1			−4.060	*p* < 0.001

**FIGURE 4:**
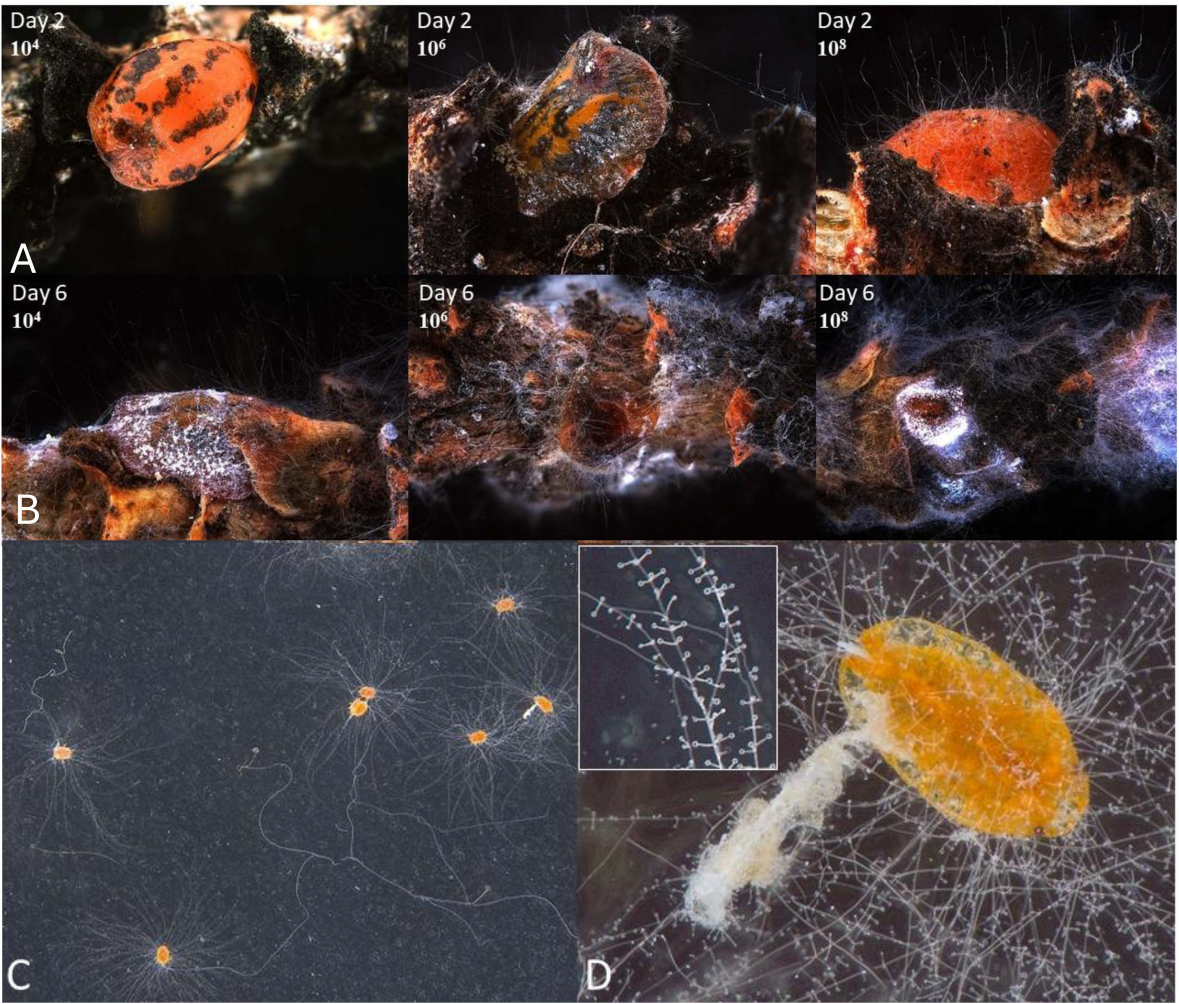
Progression of the infection of *Akanthomyces muscarius* on *Toumeyella parvicornis* adult females. The figure reports the effects of the different tested conidial concentrations from the second (A) to the sixth day (B) after the inoculation. (C) Discoloration and mycelium covering caused by *A. muscarius* on *T. parvicornis* crawlers. (D) Details of a single nymph consistently infected.

The infection progression of *T. parvicornis* adult females by *A. muscarius* (Figure 4A) showed an analogous trend over time at concentrations of 10^6^ and 10^8^, while a lower and slower trend was observed at 10^4^. As expected, the untreated control showed a zero value for the overall duration of the observations. The detailed statistics, day by day, on the comparison among the different conidial concentrations are provided in Supporting Information S3.

**FIGURE 5:**
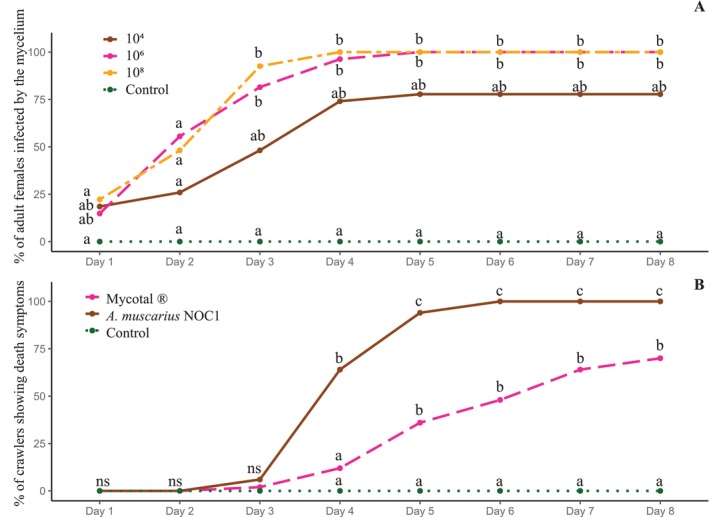
(A) Daily colonization rate of *Akanthomyces muscarius* NOC1 growing on *Toumeyella parvicornis* adult females. The colored lines indicate the different trends assessed in the different conidial concentrations and in the untreated control: 10^4^ (brown), 10^6^ (pink), 10^8^ (orange), and untreated control (green). (B) Daily colonization rate of fungi growing on *Toumeyella parvicornis* crawlers. The colored lines indicate the different rates assessed in the different tested formulations and in the untreated control: Mycotal (pink), *A. muscarius* NOC1 (brown), and untreated control (green). Different letters indicate significant differences assessed by a (GLM) followed by the Dunnett *post hoc* test (α < 0.05).

In detail, 100% of the adult females have been colonized after four and 5 days under conidial concentrations of 10^8^ and 10^6^, 78% of the specimens after 8 days under conidial concentrations of 10^4^ (Figure 5A,B).

Three days after the inoculation, 100% of the crawlers treated with *A. muscarius* showed hyphae on the body, a high percentage compared with the other treatments (38% of the crawlers colonized by Mycotal and none by the untreated control). The first group of dead crawlers was observed from the third day on, when the discoloration and complete mycelial coverage of the body became visible (Figure 4C,D). Performance of *A. muscarius* NOC1 was considerably better than Mycotal and the untreated control (Table 2).

The infection rate of the crawlers followed a similar trend for the first 3 days, after which *A. muscarius* determined, with statistical differences, more dead crawlers than Mycotal and untreated control (Figure 5B). The detail, day by day, of the comparison among the tested formulations is provided in Supporting Information S4.

### 3.5 Pathogenicity Bioassay: In Vivo Tests Simulating Natural Conditions on Overwintering *T. parvicornis* Females

The first evidence of mycelium growth on adult female specimens was detected 4 days after the treatment on stone pine twigs. Twigs treated with *A. muscarius* NOC1 strain showed abundant fungal hyphae emerging from the body. Regarding percentage, *A. muscarius* NOC1 colonized 94.8% of the treated specimens, while 2.3% were colonized after the Mycotal treatment.

Considering the complete dataset, statistical differences have been observed between the two treatments, *A. muscarius* NOC1 and Mycotal (GLM, *t. ratio* = 21.125, *p* < 0.0001). Statistical differences were also observed comparing *A. muscarius* NOC1 vs. untreated control (GLM, *t. ratio* = 21.695, *p* < 0.0001). However, no statistical differences were observed between Mycotal and untreated control (GLM, *t. ratio* = −0.570, *p* = 0.86). Mortality of the specimens, assessed at the end of the experimentation, showed the same pattern among the treatments. In fact, statistical differences were observed between *A. muscarius* NOC1 (100%) and Mycotal (25.9%) (GLM, *t. ratio* = 9.558, *p* < 0.0001). Statistical differences were also observed between *A. muscarius* NOC1 and the untreated control (23.9%) (GLM, *t. ratio* = 9.711, *p* < 0.0001). However, no statistical differences were observed between Mycotal and untreated control (GLM, *t. ratio* = −0.220, *p* = 0.98).

**FIGURE 6:**
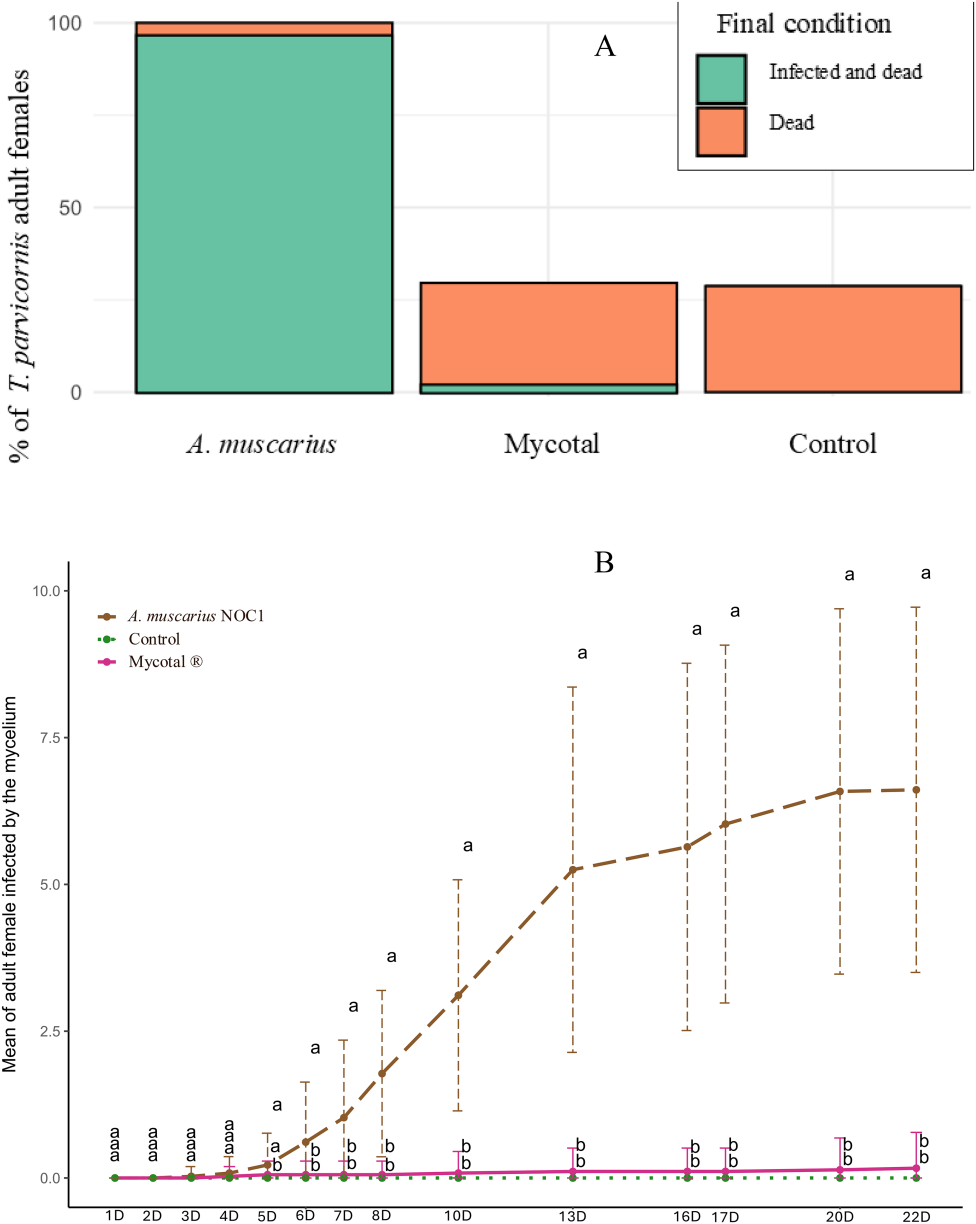
(A) Percentage of dead specimens (orange) and portion of infected and dead specimens (green) at the end of the experimentation for the different tested treatments. (B) Daily colonization of *Akanthomyces muscarius* NOC1 growing on *Toumeyella parvicornis* adult females. The whiskers indicate the standard errors. The colored lines indicate the different rates assessed in the different tested formulations and in the untreated control: Mycotal (pink), *A. muscarius* (brown), and untreated control (green). Within each sampling date, statistical differences between *A. muscarius* NOC1 and the other treatments, assessed through a (GLM) followed by the Dunnett *post hoc* test (α < 0.05), were reported with symbols *, **, ***, and NS indicating 0.05 ≤ *p* ≤ 0.01, 0.01 < *p* ≤ 0.001, *p* < 0.001, and *p* > 0.05, respectively.

In terms of percentage, 100% of the specimens, among those infected by *A. muscarius* NOC1, resulted in death at the end of the experimentation, while only 9% of the specimens, infected by Mycotal (*Lecanicillium muscarium* Ve6), were dead (Figure 6A).

The day‐by‐day statistical analysis highlighted that since the fifth day after the treatment, statistical differences started to occur (Figure 6B). From the first day until the fourth day, no statistical differences between the treatments were detected (*p* > 0.05). On the fifth day, there were statistical differences between *A. muscarius* NOC1 and untreated control (GLM, *t. ratio* 2.776, 0.05 ≤ *p* ≤ 0.01). No statistical differences were observed between = *A. muscarius* NOC1 and Mycotal (GLM, *t. ratio* = 2.082, *p* = 0.10) or between Mycotal and untreated control (GLM, *t. ratio* = −0.694, *p* = 0.80). By the sixth day, statistical differences were observed until the end of the experimentation between *A. muscarius* NOC1, Mycotal, and untreated control (*p* < 0.001).

## 4 Discussion

This study investigated, for the first time, the potential effect that different concentrations of *Akanthomyces muscarius* have on *Toumeyella parvicornis* and a first comparison with a commercial product containing entomopathogenic fungi (Mycotal, *Lecanicillium muscarium* Ve6). Besides the results on pest control, this work further confirms the presence of this entomopathogenic fungus in natural environments and its activity on *Parthenolecanium corni*, a second insect pest belonging to Coccidae. Given the limited literature describing the effects of *A. muscarius* on Coccidae (Lopes et al. 2023), this study provided a relevant contribution to a topic that is becoming pivotal in plant protection planning.

*T. parvicornis* is rapidly spreading in Europe because of the lack of efficient natural enemies as well. Autochthonous predators and parasitoids that are known to feed/develop on species genetically close to the new invaders need time to adapt and to identify the new organism as a potential prey/host (Carlsson et al. 2009; Giannetti et al. 2022; Sax and Brown 2000). Accordingly, exploring the potential pathogenicity of different autochthonous natural enemies, such as fungi, might speed up the identification of an effective control strategy that reduces the spread of this pest. This basic concept was the main driving idea of this study: the first isolation of *A. muscarius* NOC1 was carried out on a species genetically close to *T. parvicornis* , leading us to set up a dedicated investigation.

Although the *Akanthomyces* genus is taxonomically distinct from other common entomopathogenic fungi such as *Beauveria* or *Cordyceps* (Kepler et al. 2017), some of its teleomorphs are acknowledged as part of *Cordyceps* (e.g., *Cordyceps confragosa* (Mains) G.H. Sung, J.M. Sung, Hywel‐Jones & Spatafora) (Vinit et al. 2018). Our phylogenetic analysis, in accordance with Vinit et al. (2018), confirmed that *A. muscarius* NOC1 is clearly included in the *A. muscarius* cluster. The cluster also contains *Lecanicillium uredinophilum* M.J. Park, S.B. Hong & H.D. Shin, another entomopathogenic fungus mainly active on Hemiptera (Manfrino et al. 2022). A recent genomic characterization of the entomopathogenic features carried by another isolate of *A. muscarius* further confirmed its potential application as a biocontrol agent (Turco, Brugneti, et al. 2024).

The bioassay tests underlined the potential of *A. muscarius* NOC1 as a biological control candidate for *T. parvicornis* , even if further studies are necessary.

Furthermore, the preliminary observations showed that nymphs were affected by the fungus and, for a limited amount of time; they were able to walk and interact with the neighboring individuals. This fact deserves further investigation, but as the crawlers are responsible for the diffusion of the species (Malumphy et al. 2016), they might spread the fungus as well, passively contributing to the control activity (Roy et al. 2006). Moreover, it is well known that entomopathogenic fungi may induce changes in the behavior of their hosts (Roy et al. 2006; Baverstock et al. 2010; Shang et al. 2015), such as modification of their eusociality and/or reproductive and feeding attitudes. An analysis of these aspects in the case of *T. parvicornis* would strongly enhance, in combination with field studies, the role of *A. muscarius* as a biocontrol agent of this pest.

The bioassays led us to observe the development of the mycelium and the accumulation of fungal structures towards the external part of the insects' bodies. This peculiarity can be associated with the penetration mechanism of *A. muscarius* into the host, as already observed on *Ceroplastes japonicus* Green or on *Coccus hesperidum* L. (Hemiptera: Coccidae) by Liu et al. (2009, 2011). Notably, the authors proved that the penetration of the fungus mainly occurs through the anus, vulva, spiracles, or stigmatic furrow, explaining the higher accumulation of the fungal structures in the marginal part of the body.

The pathogenicity of *A. muscarius* NOC1 on adult females and nymphs of *T. parvicornis* showed an overall better performance than the commercial formulation Mycotal. Besides its pathogenicity, the trials provided an idea of the time needed by the fungus to develop in its host. The time between the infection and the evidence of the symptoms aligns with the existing literature. For instance, similar results were obtained by Liu et al. (2014), who tested the pathogenicity of *A. muscarius* on *Matsucoccus matsumurae* (Kuwana, 1905) (Hemiptera: Matsucoccidae) and *C. japonicus* .

Late autumn or early spring are seasons featured by high humidity and temperature conditions suitable enough for the development of *A. muscarius* , when overwintering females and crawlers (early spring) are mostly present in the field. Moreover, late autumn is featured by an overall lower population density of *T. parvicornis* , if compared to the rest of the year (Garonna et al. 2018); these conditions are favorable from a pest control point of view and were among the main motivations behind the preliminary observations. A conidial suspension between 10^7^ and 10^8^ was observed as the best concentration to target overwintering females, and this result is also in accordance with Muslim and AL‐Zurfi (Muslim & Al‐Zurfi 2019), who carried out tests on *Tribolium castaneum* (Herbst, 1797) (Coleoptera: Tenebrionidae), and with Sardar‐Abadi et al. (2022), who carried out tests on *Phenacoccus solenopsis* Tinsely (Hemiptera: Pseudococcidae). During the preliminary observations, after 4 days, all the females treated with a conidial concentration of 10^8^ were already covered by the mycelium, while the same condition was reached 1 day after (5 days) for the 10^6^ conidial concentration treatment. Our results are in line with Er et al. (2007) as well, who tested the pathogenicity of a *Verticillium lecanii* against *T. pityocampa* but with a lower concentration in the fungal suspension; the overall mortality of the specimens caused by NOC1 and the fungal growth on the bodies is higher. A similar test on *T. pityocampa* conducted with another strain of *A. muscarius* by Saidi et al. (2023) showed different results, but the growing humidity during the test was consistently higher than our conditions. Humidity could be a limiting factor for tests in laboratory environments and is crucial for fungal development. This aspect was highlighted by studies carried out on *Paecilomyces fumosoroseus* (Wize) A.H.S. Br. & G. Sm and *Bemisia tabaci* (Landa et al. 1994), but the knowledge of its effects, under controlled conditions, on different life stages of *T. parvicornis* can advise further field trials.

The potential use of *A. muscarius* as a biocontrol agent is promising also because of its endophytism, which has been proven on different host plants, such as *C. avellana* (Mazzaglia et al. 2023; Nicoletti and Becchimanzi 2020). There are no indications of endophytism in *P. pinea* , but further investigations should be carried out to collect this relevant information. If proven, endophytism can provide additional chances for the fungus to be effective, potentially reducing the number of treatments necessary to reach an optimal conidial concentration in the field. A further potential application, in the light of the technical difficulties involved in a classical spraying action in the open field condition, could also be the use of an attract and infect technique, which at the same time would allow greater precision of inoculation and a smaller amount of product used (Klein and Lacey 1999; Lugendo et al. 2025). It is also worth emphasizing the peculiar hyphal development of the fungus, that is, completely enveloping the body of infected individuals. This behavior, especially towards adults and gravid females, may help prevent the escape of newborn nymphs, as they could be immediately trapped in the complex web of hyphae around the individual (as observed during our trials).

As already highlighted by other studies on this topic (Di Sora et al. 2022; Di Sora, Mannu, et al. 2023; Di Sora, Rossini, et al. 2023; Di Sora, Turco, et al. 2023), *P. pinea* is still a relevant plant in Europe. This plant is part of the architecture and landscape of many Mediterranean cities, and it is cultivated for pine nut production, especially in the Iberian Peninsula (Calama et al. 2020). Preserving stone pines is a relevant and challenging action that should be carried out because *T. parvicornis* is only the last adversity of a long list.

Unfortunately, it is known that stone pine trees are susceptible to many abiotic and biotic factors (Lloret et al. 2002; Gallego et al. 2004), impacting the productivity and overall health of the plants. An example at hand is the effects of drought and of extremely high temperatures (Mutke et al. 2019), of pathogens such as *Fusarium circinatum* Nirenberg & O'Donnell (Carlucci et al. 2007), or of pests such as *Leptoglossus occidentalis* (Hemiptera: Coreidae) that directly affect the marketability of pine nuts (Farinha et al. 2021).

We believe that the results of this study open an additional door towards a sustainable and effective control of *T. parvicornis* . The next step should be a test in a more natural environment, as suggested by Gouli et al. (2013), also in combination with other control methods, ranging from endotherapic treatments to other natural enemies that will be proven to be active on this pest species.

## Author Contributions

Conceptualization, N.D.S., S.T., F.B., A.M., M.C., S.S., and L.R.; methodology, N.D.S., S.T., F.B., and A.M.; software, N.D.S., S.T., F.B., and L.R.; validation, N.D.S., S.T., F.B., and F.I.; formal analysis, N.D.S., S.T., and F.I.; investigation, N.D.S. and S.T., F.B., and F.I.; resources, A.M., M.C., S.S., and L.R.; data curation, N.D.S., S.T., M.C., and L.R.; writing – original draft preparation, N.D.S., S.T., F.B., A.M., and L.R.; writing – review and editing, N.D.S., S.T., F.B., F.I., A.M., M.C., S.S., and L.R.; visualization, N.D.S., S.T., A.M., and L.R.; supervision, A.M., M.C., S.S., and L.R.; project administration, A.M., S.S., and L.R.; funding acquisition, A.M. and S.S. All authors have read and agreed to the published version of the manuscript.

## Conflicts of Interest

The authors declare no conflicts of interest.

## Supporting information


**Data S1:** Supporting Information.

**Data S2:** Supporting Information.

**Data S3:** Supporting Information.

**Data S4:** Supporting Information.

**Table S1:** List of the primers and PCR protocol used for the amplification of six genes of *Akanthomyces muscarius*: ITS, LSU, SSU, EF‐1α, RPB1, and RPB2.
**Table S2:** *A. muscarius* NOC1 comparison to genomes of entomopathogenic fungi belonging to either *Akanthomyces, Lecanicillium* or *Simplicillium* genera, and from few representatives of *Beauveria, Cordyceps, and Salmoniella*.

## Data Availability

The data associated with this publication, as well as the R script to reproduce the results, are available at the link: https://github.com/nicodisora/aka.
