# Supplementary figures and images for: “The Last of Them”: Entomopathogenic Effect of Akanthomyces muscarius on the Scale Insect Pest Toumeyella parvicornis Under Laboratory Conditions, a Potential Biological Control Candidate
Source: Physiol Plant. 2025 Sep 20;177(5):e70533. doi: 10.1111/ppl.70533 (PMC12449707; doi:10.1111/ppl.70533)

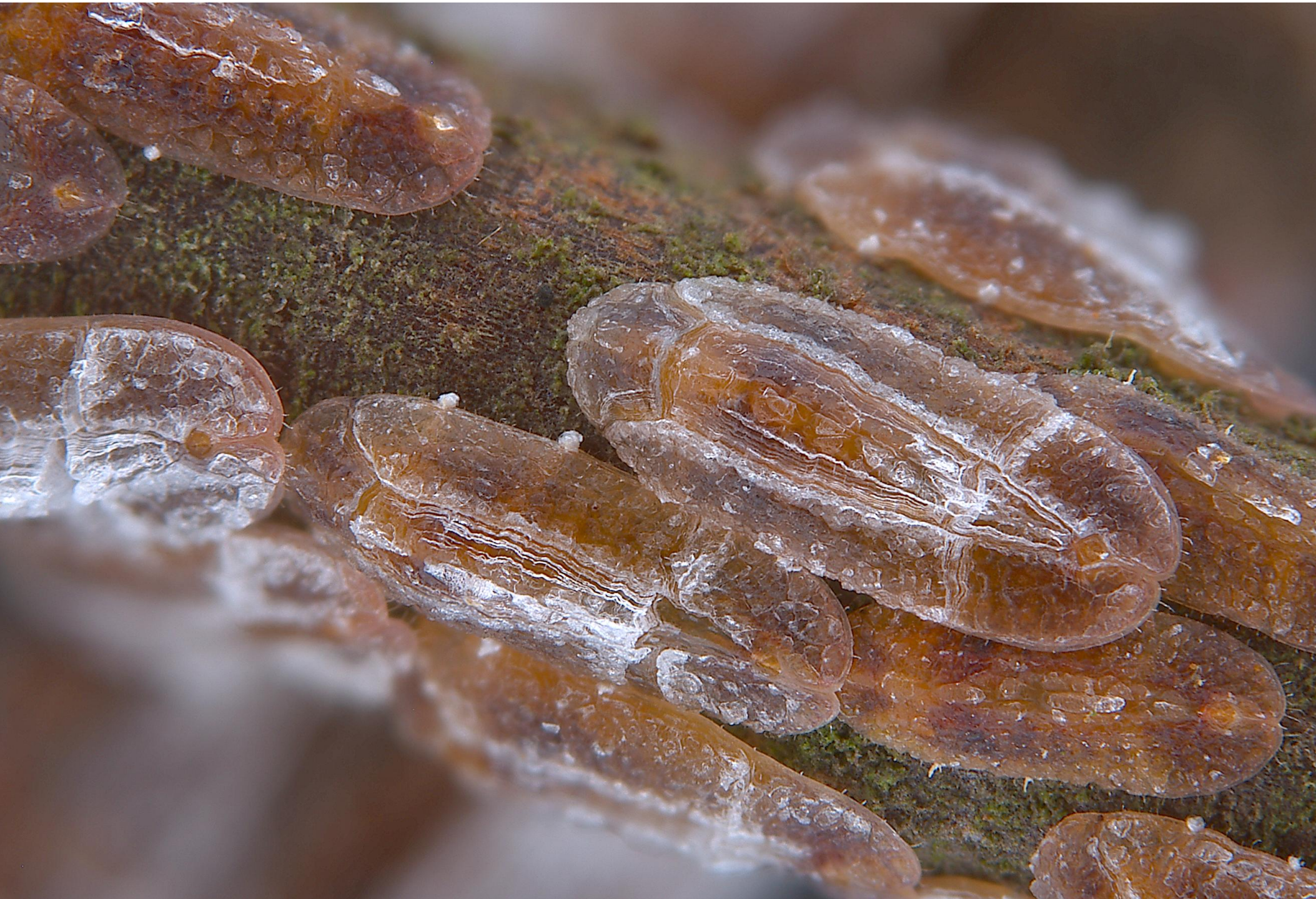

Supplement: Supplementary file 1 — Data S1: Supporting Information. [file PPL-177-e70533-s005.pdf]

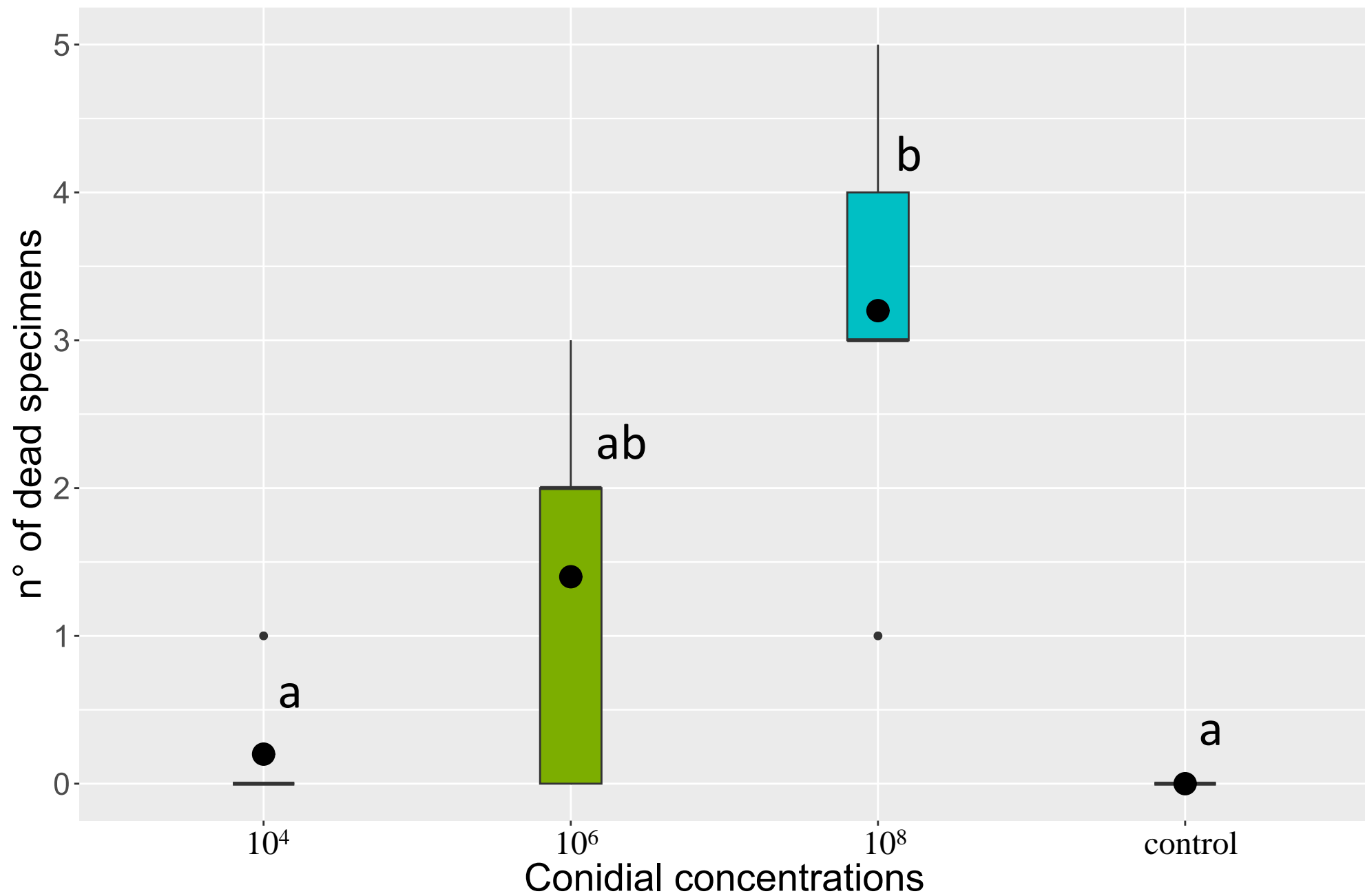

Supplement: Supplementary file 2 — Data S2: Supporting Information. [file PPL-177-e70533-s004.pdf]
