# Supplementary material for: “The Last of Them”: Entomopathogenic Effect of Akanthomyces muscarius on the Scale Insect Pest Toumeyella parvicornis Under Laboratory Conditions, a Potential Biological Control Candidate
Source: Physiol Plant. 2025 Sep 20;177(5):e70533. doi: 10.1111/ppl.70533 (PMC12449707; doi:10.1111/ppl.70533)
Supplement: Supplementary file 3 — Data S3: Supporting Information. [file PPL-177-e70533-s003.pdf]

overwintering adult females test results, day by day

DAY 1

contrast	estimate	SE	df	t.ratio	p.value
10^4 - 10^6	0.2	0.374	16	0.535	0.9571
10^4 - 10^8	-0.2	0.374	16	-0.535	0.9571
10^4 - control	1.0	0.374	16	2.673	0.0755
10^6 - 10^8	-0.4	0.374	16	-1.069	0.7410
10^6 - control	0.8	0.374	16	2.138	0.1956
10^8 - control	1.2	0.374	16	3.207	0.0266

P value adjustment: dunnettx method for 6 tests

trattamento	emmean	SE	df	lower.CL	upper.CL	.group
control	0.0	0.265	16	-0.7216	0.722	a
10^6	0.8	0.265	16	0.0784	1.522	ab
10^4	1.0	0.265	16	0.2784	1.722	ab
10^8	1.2	0.265	16	0.4784	1.922	b

DAY 2

contrast	estimate	SE	df	t.ratio	p.value
10^4 - 10^6	-1.6	1.05	16	-1.519	0.4780
10^4 - 10^8	-1.2	1.05	16	-1.139	0.7015
10^4 - control	1.4	1.05	16	1.329	0.5896
10^6 - 10^8	0.4	1.05	16	0.380	0.9838
10^6 - control	3.0	1.05	16	2.847	0.0541
10^8 - control	2.6	1.05	16	2.468	0.1103

P value adjustment: dunnettx method for 6 tests

trattamento	emmean	SE	df	lower.CL	upper.CL	.group
control	0.0	0.745	16	-2.032	2.03	a
10^4	1.4	0.745	16	-0.632	3.43	a
10^8	2.6	0.745	16	0.568	4.63	a
10^6	3.0	0.745	16	0.968	5.03	a

DAY 3

contrast	estimate	SE	df	t.ratio	p.value

```
 10^4 - 10^6        -1.8 1.19 16  -1.516  0.4796
 10^4 - 10^8        -2.4 1.19 16  -2.021  0.2364
 10^4 - control      2.6 1.19 16   2.190  0.1795
 10^6 - 10^8        -0.6 1.19 16  -0.505  0.9634
 10^6 - control      4.4 1.19 16   3.705  0.0097
 10^8 - control      5.0 1.19 16   4.211  0.0034

P value adjustment: dunnettx method for 6 tests

trattamento emmean   SE df lower.CL upper.CL .group
 control        0.0 0.84 16    -2.29     2.29  a
 10^4           2.6 0.84 16     0.31     4.89  ab
 10^6           4.4 0.84 16     2.11     6.69   b
 10^8           5.0 0.84 16     2.71     7.29   b
```

DAY 4

```
contrast       estimate   SE df t.ratio p.value
 10^4 - 10^6        -1.2 1.75 16  -0.687  0.9145
 10^4 - 10^8        -1.4 1.75 16  -0.802  0.8718
 10^4 - control      4.0 1.75 16   2.290  0.1511
 10^6 - 10^8        -0.2 1.75 16  -0.115  0.9995
 10^6 - control      5.2 1.75 16   2.978  0.0420
 10^8 - control      5.4 1.75 16   3.092  0.0335

P value adjustment: dunnettx method for 6 tests

trattamento emmean   SE df lower.CL upper.CL .group
 control        0.0 1.23 16   -3.368     3.37  a
 10^4           4.0 1.23 16    0.632     7.37  ab
 10^6           5.2 1.23 16    1.832     8.57   b
 10^8           5.4 1.23 16    2.032     8.77   b
```

DAY 5

```
contrast       estimate   SE df t.ratio p.value
 10^4 - 10^6        -1.2 1.85 16  -0.648  0.9271
 10^4 - 10^8        -1.2 1.85 16  -0.648  0.9271
 10^4 - control      4.2 1.85 16   2.268  0.1571
 10^6 - 10^8         0.0 1.85 16   0.000  1.0000
 10^6 - control      5.4 1.85 16   2.916  0.0474
 10^8 - control      5.4 1.85 16   2.916  0.0474
```

P value adjustment: dunnettx method for 6 tests

trattamento	emmean	SE	df	lower.CL	upper.CL	.group
control	0.0	1.31	16	-3.572	3.57	a
10^4	4.2	1.31	16	0.628	7.77	ab
10^6	5.4	1.31	16	1.828	8.97	b
10^8	5.4	1.31	16	1.828	8.97	b

DAY 6

contrast	estimate	SE	df	t.ratio	p.value
10^4 - 10^6	-1.2	1.85	16	-0.648	0.9271
10^4 - 10^8	-1.2	1.85	16	-0.648	0.9271
10^4 - control	4.2	1.85	16	2.268	0.1571
10^6 - 10^8	0.0	1.85	16	0.000	1.0000
10^6 - control	5.4	1.85	16	2.916	0.0474
10^8 - control	5.4	1.85	16	2.916	0.0474

P value adjustment: dunnettx method for 6 tests

trattamento	emmean	SE	df	lower.CL	upper.CL	.group
control	0.0	1.31	16	-3.572	3.57	a
10^4	4.2	1.31	16	0.628	7.77	ab
10^6	5.4	1.31	16	1.828	8.97	b
10^8	5.4	1.31	16	1.828	8.97	b

DAY 7

contrast	estimate	SE	df	t.ratio	p.value
10^4 - 10^6	-1.2	1.85	16	-0.648	0.9271
10^4 - 10^8	-1.2	1.85	16	-0.648	0.9271
10^4 - control	4.2	1.85	16	2.268	0.1571
10^6 - 10^8	0.0	1.85	16	0.000	1.0000
10^6 - control	5.4	1.85	16	2.916	0.0474
10^8 - control	5.4	1.85	16	2.916	0.0474

P value adjustment: dunnettx method for 6 tests

trattamento	emmean	SE	df	lower.CL	upper.CL	.group
control	0.0	1.31	16	-3.572	3.57	a
10^4	4.2	1.31	16	0.628	7.77	ab

10^6	5.4	1.31	16	1.828	8.97	b
10^8	5.4	1.31	16	1.828	8.97	b

DAY 8

contrast	estimate	SE	df	t.ratio	p.value
10^4 - 10^6	-1.2	1.85	16	-0.648	0.9271
10^4 - 10^8	-1.2	1.85	16	-0.648	0.9271
10^4 - control	4.2	1.85	16	2.268	0.1571
10^6 - 10^8	0.0	1.85	16	0.000	1.0000
10^6 - control	5.4	1.85	16	2.916	0.0474
10^8 - control	5.4	1.85	16	2.916	0.0474

P value adjustment: dunnettx method for 6 tests

trattamento	emmean	SE	df	lower.CL	upper.CL	.group
control	0.0	1.31	16	-3.572	3.57	a
10^4	4.2	1.31	16	0.628	7.77	ab
10^6	5.4	1.31	16	1.828	8.97	b
10^8	5.4	1.31	16	1.828	8.97	b
