# Supplementary material for: “The Last of Them”: Entomopathogenic Effect of Akanthomyces muscarius on the Scale Insect Pest Toumeyella parvicornis Under Laboratory Conditions, a Potential Biological Control Candidate
Source: Physiol Plant. 2025 Sep 20;177(5):e70533. doi: 10.1111/ppl.70533 (PMC12449707; doi:10.1111/ppl.70533)
Supplement: Supplementary file 4 — Data S4: Supporting Information. [file PPL-177-e70533-s001.pdf]

Crawlers test results, day by day

DAY 1

contrast	estimate	SE	df	t.ratio	p.value
aka - control	0	0	12	NaN	NaN
aka - mycotal	0	0	12	NaN	NaN
control - mycotal	0	0	12	NaN	NaN

P value adjustment: dunnettx method for 1 tests

trattamento	emmean	SE	df	lower.CL	upper.CL	.group
aka	0	0	12	0	0	a
control	0	0	12	0	0	a
mycotal	0	0	12	0	0	a

DAY 2

contrast	estimate	SE	df	t.ratio	p.value
aka - control	0	0	12	NaN	NaN
aka - mycotal	0	0	12	NaN	NaN
control - mycotal	0	0	12	NaN	NaN

P value adjustment: dunnettx method for 1 tests

trattamento	emmean	SE	df	lower.CL	upper.CL	.group
aka	0	0	12	0	0	a
control	0	0	12	0	0	a
mycotal	0	0	12	0	0	a

DAY 3

contrast	estimate	SE	df	t.ratio	p.value
aka - control	0.6	0.258	12	2.324	0.0975
aka - mycotal	0.4	0.258	12	1.549	0.3294
control - mycotal	-0.2	0.258	12	-0.775	0.7623

P value adjustment: dunnettx method for 3 tests

trattamento	emmean	SE	df	lower.CL	upper.CL	.group
control	0.0	0.183	12	-0.494	0.494	a
mycotal	0.2	0.183	12	-0.294	0.694	a
aka	0.6	0.183	12	0.106	1.094	a

DAY 4

contrast	estimate	SE	df	t.ratio	p.value
aka - control	6.4	0.516	12	12.394	<.0001
aka - mycotal	5.2	0.516	12	10.070	<.0001
control - mycotal	-1.2	0.516	12	-2.324	0.0975

P value adjustment: dunnettx method for 3 tests

trattamento	emmean	SE	df	lower.CL	upper.CL	.group
control	0.0	0.365	12	-0.988	0.988	a
mycotal	1.2	0.365	12	0.212	2.188	a
aka	6.4	0.365	12	5.412	7.388	b

DAY 5

contrast	estimate	SE	df	t.ratio	p.value
aka - control	9.4	1.01	12	9.338	<.0001
aka - mycotal	5.8	1.01	12	5.762	0.0003
control - mycotal	-3.6	1.01	12	-3.576	0.0104

P value adjustment: dunnettx method for 3 tests

trattamento	emmean	SE	df	lower.CL	upper.CL	.group
control	0.0	0.712	12	-1.93	1.93	a
mycotal	3.6	0.712	12	1.67	5.53	b
aka	9.4	0.712	12	7.47	11.33	c

DAY 6

contrast	estimate	SE	df	t.ratio	p.value
aka - control	10.0	0.702	12	14.237	<.0001
aka - mycotal	5.2	0.702	12	7.403	<.0001
control - mycotal	-4.8	0.702	12	-6.834	0.0001

P value adjustment: dunnettx method for 3 tests

trattamento	emmean	SE	df	lower.CL	upper.CL	.group
control	0.0	0.497	12	-1.34	1.34	a
mycotal	4.8	0.497	12	3.46	6.14	b
aka	10.0	0.497	12	8.66	11.34	c

DAY 7

contrast	estimate	SE	df	t.ratio	p.value
aka - control	10.0	0.841	12	11.896	<.0001
aka - mycotal	3.6	0.841	12	4.282	0.0029
control - mycotal	-6.4	0.841	12	-7.613	<.0001

P value adjustment: dunnettx method for 3 tests

trattamento	emmean	SE	df	lower.CL	upper.CL	.group
control	0.0	0.594	12	-1.61	1.61	a
mycotal	6.4	0.594	12	4.79	8.01	b
aka	10.0	0.594	12	8.39	11.61	c

DAY 8

contrast	estimate	SE	df	t.ratio	p.value
aka - control	10	0.683	12	14.639	<.0001
aka - mycotal	3	0.683	12	4.392	0.0024
control - mycotal	-7	0.683	12	-10.247	<.0001

P value adjustment: dunnettx method for 3 tests

trattamento	emmean	SE	df	lower.CL	upper.CL	.group
control	0	0.483	12	-1.31	1.31	a
mycotal	7	0.483	12	5.69	8.31	b
aka	10	0.483	12	8.69	11.31	c
