# Supplementary material for: “The Last of Them”: Entomopathogenic Effect of Akanthomyces muscarius on the Scale Insect Pest Toumeyella parvicornis Under Laboratory Conditions, a Potential Biological Control Candidate
Source: Physiol Plant. 2025 Sep 20;177(5):e70533. doi: 10.1111/ppl.70533 (PMC12449707; doi:10.1111/ppl.70533)
Supplement: Supplementary file 5 — Table S1: List of the primers and PCR protocol used for the amplification of six genes of Akanthomyces muscarius: ITS, LSU, SSU, EF‐1α, RPB1, and RPB2. Table S2: A. muscarius NOC1 comparison to genomes of entomopathogenic fungi belonging to either Akanthomyces, Lecanicillium or Simplicillium genera, and from few representatives of Beauveria, Cordyceps, and Salmoniella. [file PPL-177-e70533-s002.zip › Table S1.docx]

**Table S1.** List of the primers and PCR protocol used for the amplification of six genes of *Akanthomyces muscarius*: ITS, LSU, SSU, EF-1α, RPB1, and RPB2.

| **Target region** | **Primers** | **Sequence 5’-3’** | **Reference** | **PCR program** |
| --- | --- | --- | --- | --- |
| ITS | ITS1  ITS4 | TCCGTAGGTGAACCTGCGG  TCCTCCGCTTA TTGATATGC | (White et al., 1990) | 94 °C for 3 min,  35 cycles at 94 °C for 30 s, 57 °C for 30 s, 72 °C for 30 s,  72 °C for 5 min |
| SSU | NS1  NS2 | GTAGTCATATGCTTGTCTC  GGCTGCTGGCACCAGACTTGC | (White et al., 1990) | 94 °C for 4 min,  35 cycles at 94 °C for 1 min, 55 °C for 1 min, 72 °C for 90 s,  72 °C for 10 min |
| LSU | LROR  LR7 | ACCCGCTGAACTTAAGC  TACTACCACCAAGATCT | (Schneider et al., 2015) | 94 °C for 4 min,  35 cycles at 94 °C for 1 min, 47 °C for 45 s, 72 °C for 2 min,  72 °C for 10 min |
| ELF-α | EF983F  EF2218R | GCYCCYGGHCAYCGTGAYTTYATGACTTGACTTCRGTVGTGAC | (Rehner & Buckley, 2005) | 94 °C for 4 min,  35 cycles at 94 °C for 1 min, 55 °C for 1 min, 72 °C for 90 s,  72 °C for 10 min |
| RPB1 | CRPB1  RPB1Cr | CCWGGYTTYATCAAGAARGT  CCNGCDATNTCRTTRTCCATRA | (Castlebury et al., 2004) | 95 °C for 5 min,  35 cycles at 95 °C for 1 min, 47 °C for 1 min, 72 °C for 2 min,  72 °C for 5 min |
| RPB2 | RPB2-5f  bRPB2-7R2 | GAYGAYMGWGATCAYTTYGG  ACYTGRTTRTGRTCNGGRAANGG | (Liu et al., 1999) | 95 °C for 5 min,  35 cycles at 95 °C for 1 min,  55 °C for 2 min, 72 °C for 2 min, 72 C for 10 min |

**Table S2.** *A. muscarius* NOC1 comparison to genomes of entomopathogenic fungi belonging to either *Akanthomyces, Lecanicillium* or *Simplicillium* genera, and from few representatives of *Beauveria, Cordyceps, and Salmoniella*

| Assembly Accession | Organism Name | Organism Infraspecific Names Strain |
| --- | --- | --- |
| GCA_030783385.1 | *Akanthomyces dipterigenus* | MBC 701 |
| GCA_030783265.1 | *Akanthomyces dipterigenus* | MBC 708 |
| GCA_030783745.1 | *Akanthomyces dipterigenus* | MBC 691 |
| GCA_030782645.1 | *Akanthomyces dipterigenus* | MBC 628 |
| GCA_030784025.1 | *Akanthomyces dipterigenus* | MBC 741 |
| GCA_030783725.1 | *Akanthomyces dipterigenus* | MBC 690 |
| GCA_030781925.1 | *Akanthomyces dipterigenus* | MBC 678 |
| GCA_030411515.1 | *Akanthomyces dipterigenus* | MBC 259 |
| GCA_030781665.1 | *Akanthomyces dipterigenus* | MBC 608 |
| GCA_030779985.1 | *Akanthomyces dipterigenus* | MBC 561 |
| GCA_030783805.1 | *Akanthomyces dipterigenus* | MBC 685 |
| GCA_001653215.1 | *Akanthomyces lecanii* | UM487 |
| GCA_030411575.1 | *Akanthomyces lecanii* | MBC 123 |
| GCA_030780265.1 | *Akanthomyces lecanii* | MBC 542 |
| GCA_030781825.1 | *Akanthomyces lecanii* | MBC 603 |
| GCA_030772815.1 | *Akanthomyces lecanii* | MBC 159 |
| GCA_032354155.1 | *Akanthomyces lecanii* | MBC 153 |
| GCA_030785975.1 | *Akanthomyces lecanii* | MBC 801 |
| GCA_030783985.1 | *Akanthomyces lecanii* | MBC 729 |
| GCA_030789525.1 | *Akanthomyces lecanii* | MBC 901 |
| GCA_028009165.1 | *Akanthomyces muscarius* | Ve6 |
| GCA_014607475.1 | *Beauveria bassiana* | HN6 |
| GCA_001636735.1 | *Beauveria brongniartii* | RCEF 3172 |
| GCA_016490725.1 | *Beauveria felina* | SYSU-MS7908 |
| GCA_003267905.1 | *Beauveria pseudobassiana* | KACC 47484 |
| GCA_024472245.1 | *Beauveria sp.* | NWAFU-1 |
| GCA_002968875.1 | *Cordyceps cicadae* | CC02 |
| GCA_000733625.1 | *Cordyceps farinosa* | MTCC 4114 |
| GCA_003025255.1 | *Cordyceps pruinosa* | KACC 44470 |
| GCA_002591385.1 | *Cordyceps sp.* | RAO-2017 |
| GCA_003025305.1 | *Cordyceps tenuipes* | KACC 47485 |
| GCA_030411675.1 | *Lecanicillium aphanocladii* | MBC 099 |
| GCA_027595875.1 | *Lecanicillium fungicola* | Babe33 |
| GCA_900169235.1 | *Lecanicillium fungicola* | 150-1 |
| GCA_002796755.1 | *Lecanicillium psalliotae* | HWLR35 |
| GCA_027627215.1 | *Lecanicillium saksenae* | VT-O1 |
| GCA_019805375.1 | *Lecanicillium sp.* | FJII-L10 |
| GCA_002242745.2 | *Lecanicillium sp.* | LEC01 |
| GCA_003056605.1 | *Lecanicillium sp.* MT-2017a | AZ2 |
| GCA_003056565.1 | *Lecanicillium sp.* MT-2017a | CA13 |
| GCA_003056585.1 | *Lecanicillium sp.* MT-2017a | CA11 |
| GCA_023628775.1 | *Lecanicillium sp.* | S3808 |
| GCA_030778065.1 | *Lecanicillium uredinophilum* | MBC 350 |
| GCA_030783525.1 | *Lecanicillium uredinophilum* | MBC 695 |
| Wei et al., 2018 | *Lecanicillium uredinophilum* | KUN 101469 |
| Wei et al., 2018 | *Lecanicillium uredinophilum* | KUN 101466 |
| GCA_001455915.2 | *Samsoniella hepiali* | FENG |
| GCA_012273805.1 | *Simplicillium aogashimaense* | 72-15.1 |
| GCA_019843555.1 | *Simplicillium aogashimaense* | HWYR21 |
| GCA_022702485.1 | *Simplicillium sp.* | C3G150-2 |
