# Supplementary material for: “The Last of Them”: Entomopathogenic Effect of Akanthomyces muscarius on the Scale Insect Pest Toumeyella parvicornis Under Laboratory Conditions, a Potential Biological Control Candidate
Source: Physiol Plant. 2025 Sep 20;177(5):e70533. doi: 10.1111/ppl.70533 (PMC12449707; doi:10.1111/ppl.70533)
Supplement: Supplementary file 5 — Table S1: List of the primers and PCR protocol used for the amplification of six genes of Akanthomyces muscarius: ITS, LSU, SSU, EF‐1α, RPB1, and RPB2. Table S2: A. muscarius NOC1 comparison to genomes of entomopathogenic fungi belonging to either Akanthomyces, Lecanicillium or Simplicillium genera, and from few representatives of Beauveria, Cordyceps, and Salmoniella. [file PPL-177-e70533-s002.zip › ppl70533-sup-0005-TableS1@Table S1.pdf]

Assembly Accession	Organism Name	Organism Infraspecific Names Strain
GCA_030783385.1	Akanthomyces dipterigenus	MBC 701
GCA_030783265.1	Akanthomyces dipterigenus	MBC 708
GCA_030783745.1	Akanthomyces dipterigenus	MBC 691
GCA_030782645.1	Akanthomyces dipterigenus	MBC 628
GCA_030784025.1	Akanthomyces dipterigenus	MBC 741
GCA_030783725.1	Akanthomyces dipterigenus	MBC 690
GCA_030781925.1	Akanthomyces dipterigenus	MBC 678
GCA_030411515.1	Akanthomyces dipterigenus	MBC 259
GCA_030781665.1	Akanthomyces dipterigenus	MBC 608
GCA_030779985.1	Akanthomyces dipterigenus	MBC 561
GCA_030783805.1	Akanthomyces dipterigenus	MBC 685
GCA_001653215.1	Akanthomyces lecanii	UM487
GCA_030411575.1	Akanthomyces lecanii	MBC 123
GCA_030780265.1	Akanthomyces lecanii	MBC 542
GCA_030781825.1	Akanthomyces lecanii	MBC 603
GCA_030772815.1	Akanthomyces lecanii	MBC 159
GCA_032354155.1	Akanthomyces lecanii	MBC 153
GCA_030785975.1	Akanthomyces lecanii	MBC 801
GCA_030783985.1	Akanthomyces lecanii	MBC 729
GCA_030789525.1	Akanthomyces lecanii	MBC 901
GCA_028009165.1	Akanthomyces muscarius	Ve6
GCA_014607475.1	Beauveria bassiana	HN6
GCA_001636735.1	Beauveria brongniartii	RCEF 3172
GCA_016490725.1	Beauveria felina	SYSU-MS7908
GCA_003267905.1	Beauveria pseudobassiana	KACC 47484
GCA_024472245.1	Beauveria sp.	NWAFU-1
GCA_002968875.1	Cordyceps cicadae	CC02
GCA_000733625.1	Cordyceps farinosa	MTCC 4114
GCA_003025255.1	Cordyceps pruinosa	KACC 44470
GCA_002591385.1	Cordyceps sp.	RAO-2017
GCA_003025305.1	Cordyceps tenuipes	KACC 47485
GCA_030411675.1	Lecanicillium aphanocladii	MBC 099
GCA_027595875.1	Lecanicillium fungicola	Babe33
GCA_900169235.1	Lecanicillium fungicola	150-1

GCA_002796755.1	Lecanicillium psalliotae	HWLR35
GCA_027627215.1	Lecanicillium saksenae	VT-O1
GCA_019805375.1	Lecanicillium sp.	FJII-L10
GCA_002242745.2	Lecanicillium sp.	LEC01
GCA_003056605.1	Lecanicillium sp. MT-2017a	AZ2
GCA_003056565.1	Lecanicillium sp. MT-2017a	CA13
GCA_003056585.1	Lecanicillium sp. MT-2017a	CA11
GCA_023628775.1	Lecanicillium sp.	S3808
GCA_030778065.1	Lecanicillium uredinophilum	MBC 350
GCA_030783525.1	Lecanicillium uredinophilum	MBC 695
Wei et al., 2018	Lecanicillium uredinophilum	KUN 101469
Wei et al., 2018	Lecanicillium uredinophilum	KUN 101466
GCA_001455915.2	Samsoniella hepiali	FENG
GCA_012273805.1	Simplicillium aogashimaense	72-15.1
GCA_019843555.1	Simplicillium aogashimaense	HWYR21
GCA_022702485.1	Simplicillium sp.	C3G150-2
